# Variational Information Bottleneck Regularized Deep Reinforcement Learning for Efficient Robotic Skill Adaptation

**DOI:** 10.3390/s23020762

**Published:** 2023-01-09

**Authors:** Guofei Xiang, Songyi Dian, Shaofeng Du, Zhonghui Lv

**Affiliations:** 1 College of Electrical Engineering, Sichuan University, Chengdu 610065, China; 2 National Key Laboratory of Special Vehicle Design and Manufacturing Integration Technology, Baotou 014031, China

**Keywords:** reinforcement learning (RL), meta-learning, information bottleneck, deep neural networks, skill transfer, robotics

## Abstract

Deep Reinforcement Learning (DRL) algorithms have been widely studied for sequential decision-making problems, and substantial progress has been achieved, especially in autonomous robotic skill learning. However, it is always difficult to deploy DRL methods in practical safety-critical robot systems, since the training and deployment environment gap always exists, and this issue would become increasingly crucial due to the ever-changing environment. Aiming at efficiently robotic skill transferring in a dynamic environment, we present a meta-reinforcement learning algorithm based on a variational information bottleneck. More specifically, during the meta-training stage, the variational information bottleneck first has been applied to infer the complete basic tasks for the whole task space, then the maximum entropy regularized reinforcement learning framework has been used to learn the basic skills consistent with that of basic tasks. Once the training stage is completed, all of the tasks in the task space can be obtained by a nonlinear combination of the basic tasks, thus, the according skills to accomplish the tasks can also be obtained by some way of a combination of the basic skills. Empirical results on several highly nonlinear, high-dimensional robotic locomotion tasks show that the proposed variational information bottleneck regularized deep reinforcement learning algorithm can improve sample efficiency by 200–5000 times on new tasks. Furthermore, the proposed algorithm achieves substantial asymptotic performance improvement. The results indicate that the proposed meta-reinforcement learning framework makes a significant step forward to deploy the DRL-based algorithm to practical robot systems.

## 1. Introduction

Deep reinforcement learning (DRL) algorithms have achieved outstanding success in solving complex tasks, including Computer Go [1,2], manufacturing [3,4] and healthcare [5]. Generally, reinforcement learning agents learn to optimize the expected cumulative rewards by interacting with the external environment in some trial-and-error manner, which is just like how human beings learn new skills [6,7]. Therefore, DRL has received increasingly significant attention in highly nonlinear, high-dimensional robot systems for complicated tasks [8,9,10,11]. There have already emerged some impressive results in complex robotic skill learning and control, such as navigation [12,13], quadrupedal locomotion [14,15], dexterous manipulation [16], soft robots [17,18] and many more robotic tasks [19,20].

Despite the remarkable progress of the application of DRL to solve various robotic skill learning tasks, the potential assumption, i.e., the training environment and the deploy environment should be identical, results in the agents trained with DRL algorithms often over-specialize to the training environment, which is still prohibitive for adapting to some novel, unseen circumstances [21,22]. However, in modern robot application scenarios, the robot should work in an ever-changing and even unknown environment [23,24]. Intuitively, there are two methods for this problem, the first is re-training in the new environment, which would require a huge number of samples. The second is to deploy the well-trained policy directly, leading to great performance degradation, and damage to the practical robot system. Therefore, the problem of how to make the DRL agent efficiently learn and adapt to a dynamical environment is of great significance [25,26,27].

On the other hand, we deeply recognize that human beings can learn new skills for solving new tasks in new environments only through very limited samples [28]. The reason is that we can draw inferences about other cases from one instance, i.e., when new tasks emerge, we always find some related or similar cases by checking our experiences, and the skills would be abstracted and transferred to new tasks, thus we would acquire the new skills very efficiently. This kind of human intelligence gives us significant inspiration on how to improve the robot intelligence, i.e., endowing the robot with the capability of transfer learning. Many practical insights give us the possibility to realize this idea. For example, the majority of robots are not designed for a specific task, the universal robot manipulator can be used to accomplish versatile tasks and shares the same configurations and dynamics. Moreover, different tasks share the same skills, such as the door opening task and the cap unscrewing task, both of them can be decomposed into holding and rotating the object. Therefore, if we embed the mechanism of transfer learning into the DRL framework, the learning efficiency of new skills would be improved substantially.

Recently, transfer learning methods have been widely studied in the pattern recognition field [29]. The main idea of this method is that pre-training a model with huge amounts of acquired data, then fine-tune the model with a few new samples from the new task. Taylor et improving the robot intelligence, i.e., endowing the robot with the and then fine-tuning the network with few specific images [30]. Ramachandran and Howard et al. train a large long short-term memory model with the Corpora natural language processing database, then fine-tuning technique used to update the model [31]. However, this simple pre-training technique can only deal with very limited kinds of tasks, the performance is also limited by the number of new task samples. More severely, we cannot build a robot learning database like ImageNet or Corpora, so this method has hardly been applied in robotic skill transfer [32,33].

The meta-learning scheme, or learning to learn, aims to extract some similar or common structures which are shared for various tasks [34]. Once the latent common structures are obtained, one can acquire the new skills very efficiently by combining the latent structures with very few samples from the current task. Following this idea, meta-reinforcement learning [35], i.e., incorporating the meta-learning mechanism into the reinforcement learning framework, can be categorized into two classes. The first is encoding the various tasks and the according skills with memory-enabled neural networks, such as neural turning machine [36], long short term memory model, and the memorized information can be further used to assist the skill learning for new tasks [37,38]. In general, the encoding information could be the initialization parameter for new networks, and the learning parameters for new tasks. This kind of meta-learning can realize structure extraction and transfer to some extent, however, this memory-based meta-learning approach can not learn the intrinsic structure explicitly, and the learning process is like a black box.

Another kind of meta-learning approach aims to embed structure learning into policy learning. The most famous algorithm is model agnostic meta-learning (MAML) based on the policy gradient method, and can be used in nearly all policy gradient-based reinforcement learning frameworks [39,40]. The main idea of MAML is that obtain a bunch of initial parameters that are sensitive to different task gradients by formulating the parameter optimization as a bi-level optimization problem. Once the initial parameters are obtained from the source training tasks, only one or a few gradient steps are needed for the new task, then the new policy will be obtained. In comparison with the memory-based meta-learning method, MAML does not need memory-enabled neural networks, and does not introduce additional model parameters. From the perspective of feature learning, MAML can learn a bunch of task features that can be widely used for many tasks, thus a high-performance policy can be obtained through only a few policy gradients. From the dynamical system perspective, MAML hopes to find a bunch of parameters with the greatest sensitivity to all task performance. MAML has been successfully applied to practical robot learning tasks, such as the domain adaptation issue in the observing-then-imitating robot skill learning [41]. Since MAML is built on policy gradient algorithms, there still exist some issues for the computation of the policy gradient, such as the bias-variance problem in gradient estimation, and unstable gradient computation. Liu et al. proposed taming MAML algorithm to deal with the bias-variance issue by reconstructing a surrogate objective function by introducing a zero-expectation baseline function [42]. Rothfuss et al. proposed the PROMP algorithm by analyzing the credit assignment issue to improve the gradient estimation and algorithmic stability [43]. Gupta et al. studied the exploration issue and built structured noise for better exploration, thus enabling better performance [44]. However, there are still some issues, as MAML optimizes policy parameters that are most sensitive to all tasks, that is to say, the obtained policy cannot accomplish any task, including those source tasks, and once the policy adapts to one specific task, the network loses the ability of further improvement. During adaptation, all parameters of the MAML policy would be updated, and second-order optimization is involved, which aggravates the computational burden. And most of all, there is still a lack of effective methods for sub-task extraction and representation.

Inspired by how human beings adapt the obtained skill to new tasks. Pastor et al. realize the generalization of relevant features among different skills with the help of the associative skill memory technique [45,46]. Rueckert et al. use the motion primitive technique to extract a group of low-dimensional control variables that allow being reused [47]. Sutton et al. represents the process of task realization as a hierarchical motion sequence [48,49,50]. However, the aforementioned works still cannot deal with the problem of how to extract and represent the basic tasks and basic policies from the source task space, and how to infer the spatial-temporal combination of the basic tasks and basis policies automatically.

Therefore, we take advantage of latent space learning techniques and deep neural networks to automatically extract the basic tasks and basic policies, and their respective combination methods. Latent space learning techniques are widely used to compress high-dimensional data into low-dimensional information structures. Lenz et al. map the high-dimensional state space into low-dimensional latent space, and learn skills in the latent space [51]. Du et al. applies latent space to learn the dynamics of the robot, then online skill transfer is realized [52]. Generally, principal component analysis is the most classic latent space learning technique, it would struggle to deal with those high-dimensional, highly nonlinear robotic skill learning problems [53]. Tishby et al. proposed the information bottleneck technique from the perspective of information theory to extract low-dimensional structures [54]. Then, Saxe et al. extended to the deep learning domain by combining deep neural networks [55]. Alemi et al. further proposed a variational information bottleneck to quantify the inference uncertainties via variational inference theory [56]. Recent studies [57] also show that the information bottleneck principle advocates for learning minimal sufficient representations, i.e., those which contain only sufficient information for the downstream task. Optimal representations contain relevant information between input and output that is parsimonious to learn a task. Peng et al. [58] showed that variational information bottleneck could be applied to imitation learning, inverse reinforcement learning, and adversarial learning for more robust performance.

Therefore, we incorporate the variational information bottleneck into the maximum entropy off-policy reinforcement learning framework to develop a novel meta-reinforcement learning scheme. In summary, the main contributions of this article are summarized as follows:

- We develop a novel meta-reinforcement learning framework based on a variational information bottleneck. The framework consists of two stages, i.e.,  the meta-training stage and the meta-testing stage. The meta-training stage aims to extract the basic tasks and the according to basic policies, the meta-testing stage aims to efficiently infer the new policy for a new task by taking advantage of the basic tasks and basic policies.
- The meta-training and meta-testing algorithms are presented in detail. Thus, the meta-reinforcement learning framework allows efficient robotic skill transfer learning in a dynamic environment.
- Empirical experiments based on Mujoco have been conducted to show the effectiveness of the proposed scheme.

The following of this paper is organized as follows: Section 2 formally describes the problem mathematically and introduces the variational information bottleneck theory. The novel variational information bottleneck regularized DRL framework is proposed in Section 3. In Section 4, we first formulate the transfer learning tasks based on Mujoco, and then empirical results are presented. In Section 5, discussions and conclusions are represented.

## 2. Problem Formulation and Background

### 2.1. Problem Formulation

Meta-reinforcement learning involves two stages, meta-training and meta-testing. During the meta-training stage, a batch of tasks is first sampled from the source task space as the training set, then an algorithm is employed for model training. During the meta-testing stage, a small batch of tasks is sampled from the target task space as a testing set, using the well-trained model to evaluate the transfer performance through only a few interactions on each task. In general, assuming that the training task set and testing task set are sampled from the same distribution $p\left(T\right)$, and the task space consists of state space S, action space A, transition function P, and bounded reward function space R, every task could be characterized by Markov Decision Process (MDP), we further assume that the transition functions and reward functions are unknown to the agent, only samples could be gathered. Stochastically sampling a task $T=\left\{\begin{array}{ccc} p\left(s_{0}\right), & p\left(\left.s_{t+1}\right|s_{t},a_{t}\right), & r\left(s_{t},a_{t}\right) \end{array}\right\}$, where $p\left(s_{0}\right)$ denotes the initial state distribution, $p\left(\left.s_{t+1}\right|s_{t},a_{t}\right)$ denotes the transition probability, which is unknown, $r\left(s_{t},a_{t}\right)$ denotes reward function. From the aforementioned definition, $p\left(T\right)$ can describe both tasks with different transition functions, i.e., different robot dynamics, and tasks with different reward functions, i.e., different tasks, but assuming that all robots and tasks share with the same state space and action space. When given a task distribution $p\left(T\right)$, we firstly sample *M* tasks as the meta-training set $\left\{T_{i}\right\}∼p\left(T\right),\left(i=1,\cdots ,M\right)$, then we can optimize the latent space based policy $\pi \left(\left.a\right|s,z\right)$, where *z* denotes the latent space, with these training tasks. Denoting $e_{k}^{T}=\left\{s_{k},a_{k},r_{k},s_{k}^{\prime }\right\}$ as a sample for the task T, then $e_{1:K}^{T}$ denote a trajectory. Thus, we can obtain the meta-training data space $\left\{D_{T_{i}}\right\},i=1,\cdots ,M$. Once meta-training accomplished, we can sample another *N* tasks from the task distribution $p\left(T\right)$ as meta-testing set $\left\{T_{j}\right\}∼p\left(T\right),\left(j=1,\cdots ,N\right)$. For any test task, only a few interactions are needed, then the latent space can infer the spatial-temporal combination of basic tasks. Thus, the agent can adapt to the test task efficiently. The detailed variable definitions are shown in Table 1.

The latent space-based meta-reinforcement learning can simultaneously learn the latent space and its corresponding policy. The latent space is obtained in a self-supervised manner and used to differentiate various tasks. Thus, the latent space should satisfy the following principles, (1) sufficiency, the latent space *z* should sufficiently characterize the task space $p\left(T\right)$, i.e., the mapping from task space to latent space should be surjective and injective. (2) Parsimony, the latent space should not be over-fit to the detailed state. (3) Identifiability, the latent space could identify the specific task from the trajectories. Therefore, we tailored the variational information bottleneck technique to extract the aforementioned latent space. Furthermore, to achieve better training efficiency, we consider the state-of-art maximum entropy off-policy actor-critic algorithm, i.e., soft actor-critic (SAC). In the following subsections, we will describe the mathematical formulation of reinforcement learning and variational information bottleneck theory.

### 2.2. Markov Decision Process (MDP)

In general, the MDP is describe in tuple $M=\left(\begin{array}{cccccc} S, & A, & P, & r, & \gamma , & \rho_{0} \end{array}\right)$, where S, A and P:S×A×S→[0,∞) with definition before. r:S×A→R denotes the reward function, bounded by $\left[\underline{r},\bar{r}\right]$. $\rho_{0}:S\to R$ denotes the initial state distribution, and γ∈(0,1) denotes the discount factor.

In this work, we consider stochastic policy, i.e., π:S×A⟶[0,1], and let J(π) denote the expected discounted cumulative reward:

(1) $$J\left(\pi \right)=E_{s_{0},a_{0},\cdots }\left[\overset{\infty }{\underset{t=0}{\sum }}\gamma^{t}r\left(s_{t},a_{t}\right)\right],$$

where E means expectation, by taking on a bunch of trajectories following the policy π. The objective of RL is to optimize the policy by maximizing the objective function:

(2) $$\pi^{*}=\underset{\pi }{\arg\max}J\left(\pi \right),$$

where $s_{0}∼\rho_{0}\left(s_{0}\right),s_{t+1}∼P\left(\left.s_{t+1}\right|a_{t},s_{t}\right),a_{t}∼\pi \left(\left.a_{t}\right|s_{t}\right)$. Let $Q_{\pi }$ denotes the state-action value function, $V_{\pi }$ denotes the state value function, and $A_{\pi }$ denotes the advantage function:

(3) $$Q_{\pi }\left(s_{t},a_{t}\right)=E_{s_{t+1},a_{t+1},\cdots }\left[\overset{\infty }{\underset{k=0}{\sum }}\gamma^{k}r\left(s_{t+k}\right)\right],$$

(4) $$V_{\pi }\left(s_{t}\right)=E_{a_{t},s_{t+1},\cdots }\left[\overset{\infty }{\underset{k=0}{\sum }}\gamma^{k}r\left(s_{t+k}\right)\right],$$

(5) $$A_{\pi }\left(s_{t},a_{t}\right)=Q_{\pi }\left(s_{t},a_{t}\right)-V_{\pi }\left(s_{t}\right),$$

where, $a_{t}∼\pi \left(\left.a_{t}\right|s_{t}\right),s_{t+1}∼P\left(\left.s_{t+1}\right|a_{t},s_{t}\right)$ for all t≥0.

### 2.3. Maximum Entropy Actor-Critic

To balance the exploration and exploitation problem in the original RL, the per-step entropy is usually added to the original per-step reward as $r\left(s_{t},a_{t}\right)+\alpha H\left(\pi \left(\left.\cdot \right|s_{t}\right)\right)$, where $H\left(\pi \left(\left.\cdot \right|s_{t}\right)\right)=-\int_{A}\pi \left(\left.\cdot \right|s_{t}\right)\log\left(\pi \left(\left.\cdot \right|s_{t}\right)\right)da_{t}$ denotes the entropy of the policy at $s_{t}$, α∈(0,1) is a weighting term. Then, the optimal policy would be achieved by maximizing the discounted reward and its entropy in expectation:

(6) $$\begin{array}{c} \pi^{*}=\underset{\pi }{\arg\max}\overset{\infty }{\underset{t=0}{\sum }}E_{\left(s_{t},a_{t}\right)∼\rho_{\pi }}\gamma^{t}\left[\right.\left.r\left(s_{t},a_{t}\right)+\alpha H\left(\pi \left(\left.\cdot \right|s_{t}\right)\right)\right]. \end{array}$$

By doing so, the agent aims to maximize the cumulative reward, whilst keeping the policy as stochastic as possible. Several previous works have studied this framework carefully, and shown some conceptual and practical advantages [9,10,11]. The policy is incentivized to explore more diversely, while giving up those obviously hopeless directions by showing improved sampling efficiency and performance. In comparison with general RL, the action-state value function is modified as

(7) $$\begin{array}{c} Q^{*}\left(s_{t},a_{t}\right)=r\left(s_{t},a_{t}\right)+E_{\left(s_{t},a_{t}\right)∼\rho_{\pi }}\left[\overset{\infty }{\underset{k=1}{\sum }}\gamma^{k}\left(r\left(s_{t+k}\right)+\alpha H\left(\pi^{*}\left(\left.\cdot \right|s_{t+k}\right)\right)\right)\right], \end{array}$$

and the state value function as

(8) $$V^{*}\left(s_{t+1}\right)=\alpha \log\int_{A}\exp\left(\frac{1}{\alpha }Q^{*}\left(s_{t},a^{\prime }\right)\right)da^{\prime }.$$

where $\rho_{\pi }\left(s,a\right)=\pi \left(\left.a\right|s\right)\overset{\infty }{\underset{t=0}{\sum }}\gamma^{t}P\left(\left.s_{t}=s\right|\pi \right)$ and $\rho_{\pi }\left(s\right)=E_{a∼\pi \left(\left.\cdot \right|s\right)}\left[\rho_{\pi }\left(s,a\right)\right]$ denote the state-action occupancy measure and state occupancy measure, respectively. Then, according to the Bellman optimality principle, we obtain the following results.

**Lemma 1.**  *As the modified action-state value function and the modified state value function defined in Equation (7) and Equation (8), respectively, the modified action-state value function and the modified state value function satisfy the Bellman optimality equation*

(9) $$Q^{*}\left(s_{t},a_{t}\right)=r\left(s_{t},a_{t}\right)+\gamma E_{s_{t+1}∼p_{s}}\left[V^{*}\left(s_{t+1}\right)\right],$$

*with the optimal policy given by*

(10) $$\pi^{*}\left(a_{t}|s_{t}\right)=\exp\left(\frac{1}{\alpha }\left(Q^{*}\left(s_{t},a_{t}\right)-V^{*}\right)\right).$$

*Moreover, the modified value iteration is given by*

(11) $$Q\left(s_{t},a_{t}\right)\leftarrow r\left(s_{t},a_{t}\right)+\gamma E_{s_{t+1}∼p_{s}}\left[V\right],\forall s_{t},a_{t},$$

(12) $$V\left(s_{t}\right)\leftarrow \alpha \log\int_{A}\exp\left(\frac{1}{\alpha }Q\left(s_{t},a^{\prime }\right)\right)da^{\prime },\forall s_{t},$$

*converges to their fix point $Q^{*}\left(s_{t},a_{t}\right)$ and $V^{*}\left(s_{t}\right)$, respectively.*

The modified Bellman optimality equation in Equation (9) can be regarded as a generalization of the conventional Bellman equation, which could be recovered as α→0. The SQL (Soft Q-Learning) and SAC (Soft Actor-Critic) algorithms, which are built on the aforementioned value iteration, can achieve state-of-art performance in continuous control tasks and dexterous hand manipulation tasks, by using deep neural networks to approximate the modified action-value function, the modified state value function and the policy [10].

### 2.4. Variational Information Bottleneck (VIB)

The information bottleneck theory was first proposed by Tishby et al., and Alemi et al. proposed variational information bottleneck by incorporating variational inference and deep learning techniques. For a general supervised learning task, given a data set $\left\{\begin{array}{cc} x_{i}, & y_{i} \end{array}\right\}$, where $x_{i}$ denotes the input, $y_{i}$ denotes the label. Information bottleneck encodes the inputs into a latent space as $E\left(\left.z\right|x\right)$, and constrain the information flow between *x* and *z* using mutual information I(X;Z), defined by,

(13) $$\begin{array}{cc} I(Z,X) & =D_{\mathrm{KL}}\left[p\left(Z,X\right)|p\left(Z\right)p\left(x\right)\right]\\ \; & =E_{X∼p(X)}\left[D_{\mathrm{KL}}\left[E\left(Z|X\right)|p\left(Z\right)\right]\right], \end{array}$$

where $D_{KL}$ denotes Kullback-Leibler (KL) divergence.

Thus, we obtain a constrained optimization problem as,

(14) $$\begin{array}{c} J\left(q,E\right)=\min_{q,E}E_{x,y∼p\left(x,y\right)}\left[E_{z∼E\left(\left.z\right|x\right)}\left[-\log q\left(\left.y\right|z\right)\right]\right]\\ \begin{array}{cccccc} & & & & s.t. & I\left(X,Z\right)\leq \end{array}I_{c}, \end{array}$$

where $I_{c}$ denotes the user-defined information constraint, $E\left(\left.z\right|x\right)$ denotes the latent space encoder, $q\left(\left.y\right|z\right)$ denotes the mapping from latent space to the label space. By solving this constrained optimization problem, we obtain a latent space that is sufficient for the final task and parsimony enough without redundancy information.

In practice, one can take samples of $D_{\mathrm{KL}}\left[E\left(Z|X\right)|p\left(Z\right)\right]$ to estimate the mutual information. While E(Z∣X) is straightforward to compute, calculating p(Z) requires marginalization across the entire state space *S*, which is impossible since most non-trivial environments are intractable. Instead, we introduce an approximator, $q\left(Z\right)∼N\left(\vec{0},I\right)$, to replace p(Z). This achieves an upper bound on I(Z,X),

(15) $$\begin{array}{cc} \; & E_{X∼p(X)}\left[D_{\mathrm{KL}}\left[E\left(Z|X\right)|q\left(Z\right)\right]\right]\\ = & \int_{x}dxp\left(x\right)\int_{z}dzE\left(z|x\right)\log\frac{E(z|x)}{q(z)}\\ = & \int_{z,x}dxdzE\left(z|x\right)\log E\left(z|x\right)-\int_{z}dzp\left(z\right)\log q\left(z\right)\\ \geq & \int_{z,x}dxdzE\left(z|x\right)\log E\left(z|x\right)-\int_{z}dzp\left(z\right)\log p\left(z\right)\\ = & \int_{x}dxp\left(x\right)\int_{z}dzE\left(z|x\right)\log\frac{E(z|x)}{p(z)}\\ = & I(Z,X), \end{array}$$

where the inequality arises because of the non-negativeness KL-divergence, i.e., $D_{\mathrm{KL}}\left[p\left(z\right)|q\left(z\right)\right]\geq 0$.

Substituting Equation (15) into Equation (14) obtains,

(16) $$\begin{array}{c} \tilde{J}\left(q,E\right)=\min_{q,E}E_{x,y∼p\left(x,y\right)}\left[E_{z∼E\left(\left.z\right|x\right)}\left[-\log q\left(\left.y\right|z\right)\right]\right]\\ \begin{array}{cccc} & & s.t. & E_{x∼p\left(x\right)}\left[D_{KL}\left[E\left(\left.z\right|x\right)∥p\left(z\right)\right]\right]\leq \end{array}I_{c}. \end{array}$$

where $\tilde{J}\left(q,E\right)\geq J\left(q,E\right)$ denotes the upper bound of the objective function. By taking advantage of lagrangian multiplier β, we convert the constrained optimization problem into an unconstrained optimization problem,

(17) $$\min_{q,E}E_{x,y∼p\left(x,y\right)}\left[E_{z∼E\left(\left.z\right|x\right)}\left[-\log q\left(\left.y\right|z\right)\right]\right]+\beta \left(E_{x∼p\left(x\right)}\left[D_{KL}\left[E\left(\left.z\right|x\right)∥p\left(z\right)\right]\right]-I_{c}\right).$$

By solving the aforementioned problem, we can obtain a sufficient and parsimonious representation of the original data distribution. Alemi et al. showed that the variational information bottleneck approach could suppress the parameter over-fitting problem, and the obtained model was robust to adversarial attacks.

## 3. VIB Based Meta-Reinforcement Learning

### 3.1. Overview

**Figure 1:**
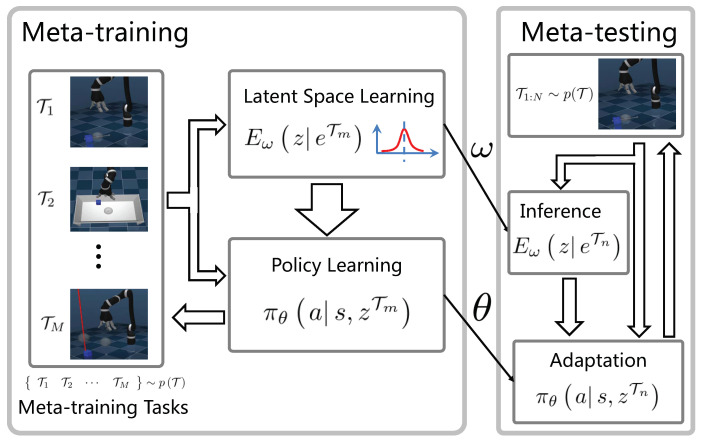
Latent space-based robotic skill transfer learning framework.

The latent space-based robotic skill transfer learning framework is shown in Figure 1, which includes two stages. During the meta-training stage, we sample from the source task space and obtain *M* training tasks, for each task, the RL agent interacts with the environment and obtains several trajectories $e_{1:K}^{T}$, then we iteratively optimize the latent space encoder $E_{\omega }\left(\left.z\right|e^{T_{m}}\right)$ and latent-based policy $\pi_{\theta }\left(\left.a\right|s,z^{T_{m}}\right)$, which both parameterized by deep neural networks with parameters as ω and θ, respectively. Once the training process converges, the two networks are fixed, and they would be reused for test tasks. During the meta-testing stage, we sample another *N* tasks, the latent space encoder infers the specific task from very few interacting samples, then the latent vector informs the policy to synthesize the final policy. During the test stage, there is no gradient update, so the agent can reuse the obtained skills, which would be very efficient. Moreover, since the latent space would inform the agent of the way to synthesize new policy according to the real-time data sequence for a specific task, improved performance would be achieved.

Following the procedure of maximum entropy actor-critic framework, the latent-based constrained optimization problem is formulated as,

(18) $$\begin{array}{c} J\left(\pi ,E\right)=\max_{\pi ,E}E_{T∼p\left(T\right)}\left[E_{z∼E\left(\left.z\right|e^{T}\right)}\left[R\left(e^{T},z\right)+\alpha H\left(\pi \left(\left.\cdot \right|s_{t},z_{t}\right)\right)\right]\right]\\ \begin{array}{cccc} \; & & s.t. & I\left(e^{T},Z\right)\leq I_{c}, \end{array} \end{array}$$

where $R\left(e^{T},z\right)$ denotes the latent space informed trajectory discounted return $e^{T}$.

According to the variational information bottleneck theory, introducing the variational lower bound, one obtains,

(19) $$\begin{array}{c} \hat{J}\left(\pi ,E\right)=\max_{\pi ,E}E_{T∼p\left(T\right)}\left[E_{z∼E\left(\left.z\right|e^{T}\right)}\left[R\left(e^{T},z\right)+\alpha H\left(\pi \left(\left.\cdot \right|s_{t},z_{t}\right)\right)\right]\right]\\ \;s.t.\;E_{T∼p\left(T\right)}\left[D_{KL}\left[E\left(\left.z\right|e^{T}\right)∥p\left(z\right)\right]\right]\leq I_{c}, \end{array}$$

where $\hat{J}\left(\pi ,E\right)$ denotes the upper bound of $J\left(\pi ,E\right)$.

According to the maximum entropy Bellman optimality principle, denote the optimal action-state value function for the problem (19) as

(20) $$\begin{array}{c} Q^{*}\left(s_{t},a_{t},z_{t}\right)=r\left(s_{t},a_{t},z_{t}\right)+\\ \begin{array}{ccc} & & \;\;\;\;E_{\left(s_{t},a_{t}\right)∼D_{T_{i}};z∼E\left(\left.z\right|e^{T}\right)}\left[\overset{\infty }{\underset{k=1}{\sum }}\gamma^{k}\left(r\left(s_{t+k},a_{t+k},z_{t+k}\right)+\alpha H\left(\pi^{*}\left(\left.\cdot \right|s_{t+k},z_{t+k}\right)\right)\right)\right], \end{array} \end{array}$$

with the optimal state value function as,

(21) $$V^{*}\left(s_{t+1},z_{t+1}\right)=\alpha \log\int_{A}\exp\left(\frac{1}{\alpha }Q^{*}\left(s_{t},a^{\prime },z\right)\right)da^{\prime }.$$

According to Lemma 1, the optimal Bellman equation is,

(22) $$B^{\pi }Q^{*}\left(s_{t},a_{t},z_{t}\right)=r\left(s_{t},a_{t},z_{t}\right)+\gamma E_{s_{t+1}∼p_{s}}\left[V^{*}\left(s_{t+1},z_{t+1}\right)\right],$$

followed, by optimal policy, as

(23) $$\pi^{*}\left(a_{t}|s_{t},z_{t}\right)=\exp\left(\frac{1}{\alpha }\left(Q^{*}\left(s_{t},a_{t},z_{t}\right)-V^{*}\left(s_{t},z_{t}\right)\right)\right).$$

For practical robotic skill learning problems, the state space and action space are always very high dimensional, so we utilize deep neural networks to approximate the action-state value function $Q_{\varphi }$, state value function $V_{\psi }$, policy $\pi_{\theta }$ and latent space encoder $E_{\omega }$ with network parameters as ψ, φ, θ, and ω, respectively.

### 3.2. Latent Space Learning

From Equation (19), the latent space modeling should simultaneously satisfy two requirements, maximizing the entropy-augmented returns for task accomplishment and the information bottleneck constraint. So, the latent learning algorithm should simultaneously minimize the Bellman residual $∥B^{\pi }Q-Q∥$ and KL divergence $D_{KL}\left[p\left(Z\right)∥p\left(Z\right)\right]\geq 0$, the cost function is formulated as,

(24) $$\begin{array}{c} J_{E_{\omega }}\left(D,z\right)=\underset{T_{i}∼p\left(T\right)}{\sum }\left(\frac{1}{2}\underset{\left(s_{t},a_{t},s_{t}\right)∼D}{\sum }\left(Q_{\phi }\left(s_{t},a_{t},z_{t}\right)-\left(r\left(s_{t},a_{t},z_{t}\right)\right.\right.\right.\\ \;\left.\;{\left.\left.\;\;+\gamma E_{s_{t+1}∼p;z_{t+1}∼E}\left[V_{\bar{\psi }}\left(s_{t+1},z_{t+1}\right)\right]\right)\right)}^{2}+\beta D_{KL}\left[E_{\omega }\left(\left.z\right|e^{T}\right)∥p\left(z\right)\right]\right), \end{array}$$

where $V_{\bar{\psi }}$ denotes the target network for algorithm stability. Taking the computation feasibility into account, implementing p(z) as Gaussian distribution, i.e., $p\left(z\right)=\prod_{i=1}^{K}p\left(z^{i}\right)=\prod_{i=1}^{K}N\left(0,I\right)$, where $N\left(0,I\right)$ is also Gaussian distributions. Thus, the $E_{\omega }$ can be modeled as multi-variable Gaussian distributions as,

(25) $$E_{\omega }\left(\left.z\right|e_{1:K}^{T}\right)\propto \prod_{k=1}^{K}N\left(\mu_{\omega }\left(e_{k}^{T}\right),\sigma_{\omega }\left(e_{k}^{T}\right)\right),$$

where, $\mu_{\omega }\left(e_{k}^{T}\right)$ and $\sigma_{\omega }\left(e_{k}^{T}\right)$ denote the mean and variance. The latent space encoder network updating rule is,

(26) $$\omega_{t+1}\leftarrow \omega_{t}-\eta_{\omega }{\hat{\nabla }}_{\omega }J_{E_{\omega }}\left(D,z\right),$$

where $\eta_{\omega }$ denotes updating rate, and ${\hat{\nabla }}_{\omega }$ denotes the first-order gradient.

Using the aforementioned Bayesian optimization protocol, we can realize skill transfer from task space to new tasks by posteriori inference. Furthermore, the intrinsic uncertainty estimation property can be used to explore a better way of task realization.

### 3.3. Vib Based Meta-Reinforcement Learning Algorithm

According to Lemma 1, we know that the Bellman equation in Equation (22) is a contraction mapping, i.e., the optimal action-state value function is the fixed point. So the cost function for the action-state value function is,

(27) $$J_{Q_{\phi }}\left(D,z\right)=\frac{1}{2}E_{\left(s_{t},a_{t},r_{t},s_{t+1}\right)∼D}\left[{\left(Q_{\phi }\left(s_{t},a_{t},z_{t}\right)-\left(r\left(s_{t},a_{t},z_{t}\right)+\gamma E_{s_{t+1}∼p}\left[V_{\bar{\psi }}\left(s_{t},z_{t}\right)\right]\right)\right)}^{2}\right].$$

And we get the action-stage value function network parameter’s updating rule by gradient descending,

(28) $$\varphi_{t+1}\leftarrow \varphi_{t}-\eta_{\varphi }{\hat{\nabla }}_{\varphi }J_{Q_{\phi }}\left(D,z\right),$$

where $\eta_{\varphi }$ denotes the learning rate, ${\hat{\nabla }}_{\varphi }$ denotes the unbiased estimation of gradient estimation.

According to Equation (21), the cost function for state value function is,

(29) $$J_{V_{\psi }}\left(D,z\right)=\frac{1}{2}E_{\left(s_{t},a_{t},r_{t},s_{t+1}\right)∼D}\left[{\left(V_{\psi }\left(s_{t},a_{t},z_{t}\right)-E_{a_{t}∼\pi_{\theta }}\left[Q_{\phi }\left(s_{t},a_{t},z_{t}\right)-\alpha \log\pi_{\theta }\left(\left.a_{t}\right|s_{t},z_{t}\right)\right]\right)}^{2}\right].$$

where the computation of the action-state value function using actions from the current policy. We get the state value function network parameter’s updating rule as,

(30) $$\psi_{t+1}\leftarrow \psi_{t}-\eta_{\psi }{\hat{\nabla }}_{\psi }J_{V_{\psi }}\left(D,z\right),$$

where $\eta_{\psi }$ denotes the learning rate, and ${\hat{\nabla }}_{\psi }$ denotes the unbiased estimation.

According to Equation (23), the cost function for policy network is,

(31) $$J_{\pi_{\theta }}\left(D,z\right)=D_{KL}\left(\pi_{\theta }\left(\left.a_{t}\right|s_{t},z_{t}\right)∥\exp\left(\frac{1}{\alpha }Q_{\phi }\left(s_{t},a_{t},z_{t}\right)\right)-\log\left(Z_{\phi }\left(s_{t},z_{t}\right)\right)\right),$$

where $Z_{\phi }\left(s_{t},z_{t}\right)$ denotes the normalizing function, which is irrelevant to the computation of policy function gradient. So the policy network parameter’s updating rule is,

(32) $$\theta_{t+1}\leftarrow \theta_{t}-\eta_{\theta }{\hat{\nabla }}_{\theta }J_{\pi_{\theta }}\left(D,z\right),$$

where $\eta_{\theta }$ denotes the learning rate, ${\hat{\nabla }}_{\theta }$ denotes the unbiased gradient estimation.

According to the aforementioned analysis, we could formulate the VIB-based meta-reinforcement learning algorithm. The meta-training protocol is presented in Algorithm 1, and the meta-testing procedure is presented in Algorithm 2. We use an ADAM optimizer for deep neural network training.
**Algorithm 1** VIB based meta-reinforcement learning training algorithm.1:**Input:**D: training data set; $\eta_{\theta },\eta_{\varphi },\eta_{\psi },\eta_{\omega }$ denote learning rate; ${\left\{T_{m}\right\}}_{m=1,\ldots ,M}∼p\left(T\right)$: meta-training task set.2:**Output:** policy network $\pi_{\theta }$ and latent space encoder $E_{\omega }$.3:Setting parameter for the target network: $\psi \leftarrow \bar{\psi }$; the initial sample set for each task $D^{m}$4:**for** *epoch* **do**5:     **for** $T_{m}$ **do**6:          Initializing each trajectory: $e^{T_{m}}=\left\{\right\}$7:          **for** k=1,⋯,N **do**8:             Latent space inference $z∼E_{\omega }\left(\left.z\right|e^{T_{m}}\right)$9:             The current policy $\pi_{\theta }\left(\left.a\right|s,z\right)$ interact with each task and obtain sample $D^{m}$10:            Updating $e^{T_{m}}={\left\{\left(s_{j},a_{j},s_{j}^{\prime },r_{j}\right)\right\}}_{j:1\ldots K}∼D^{m}$11:         **end for**12:    **end for**13:    **for** *step* **do**14:         **for** $T_{m}$ **do**15:           Sampling from the training data set: $e^{T_{m}},d^{m}∼D^{m}$16:           Latent space inference: $z∼E_{\omega }\left(\left.z\right|e^{T_{m}}\right)$17:           Computing the action-state value function: $J^{m}\left(Q\right)=J_{E_{\varphi }}\left(d^{m},z\right)$18:           Computing the state value function: $J^{m}\left(V\right)=J_{E_{\psi }}\left(d^{m},z\right)$19:           Computing the policy cost function: $J^{m}\left(\pi \right)=J_{E_{\theta }}\left(d^{m},z\right)$20:           Computing the latent space encoder cost function: $J^{m}\left(E\right)=J_{E_{\omega }}\left(d^{m},z\right)$21:         **end for**22:         Updating the action-state value function network: $\varphi_{t+1}\leftarrow \varphi_{t}-\eta_{\varphi }{\hat{\nabla }}_{\varphi }\underset{m}{\sum }J^{m}\left(Q\right)$23:         Updating the state value function network: $\psi_{t+1}\leftarrow \psi_{t}-\eta_{\psi }{\hat{\nabla }}_{\psi }\underset{m}{\sum }J^{m}\left(V\right)$24:         Updating the policy network: $\theta_{t+1}\leftarrow \theta_{t}-\eta_{\theta }{\hat{\nabla }}_{\theta }\underset{m}{\sum }J^{m}\left(\pi \right)$25:         Updating the latent space encoder network: $\omega_{t+1}\leftarrow \omega_{t}-\eta_{\omega }{\hat{\nabla }}_{\omega }\underset{m}{\sum }J^{m}\left(E\right)$26:    **end for**27:    **Updating the target network** $\psi \leftarrow \bar{\psi }$28:**end for**

**Algorithm 2** VIB based meta-reinforcement learning testing algorithm.
1:**Input:**${\left\{T_{n}\right\}}_{m=1,\ldots ,N}∼p\left(T\right)$: Meta-testing task set; θ: meta-training policy network; ω: meta-training latent space encoder network.2:Initializing the trajectory: $e^{T}=\left\{\right\}$3:**for** k=1,⋯,N **do**4:      Latent space inference: $z∼E_{\omega }\left(\left.z\right|e^{T}\right)$5:      Using current policy $\pi_{\theta }\left(\left.a\right|s,z\right)$ to interact with each task and obtain $D^{k}$6:      Sample accumulation: $e^{T}=e^{T}\cup D^{k}$7:      Evaluating empirical discounted return for each task.8:
**end for**



## 4. Experiments

Our experiments aim to investigate the following questions:

1. Does the VIB-based meta-reinforcement learning algorithm realize efficient skill transfer?
2. How about the learning efficiency and asymptotic performance of the VIB-based meta-reinforcement learning algorithm in comparison with that of other meta-learning approaches, such as MAML and ProMP?
3. Does the VIB-based meta-reinforcement learning algorithm can improve the learning performance during the training stage in comparison with other algorithms?

### 4.1. Experiments Configuration

**Figure 2:**
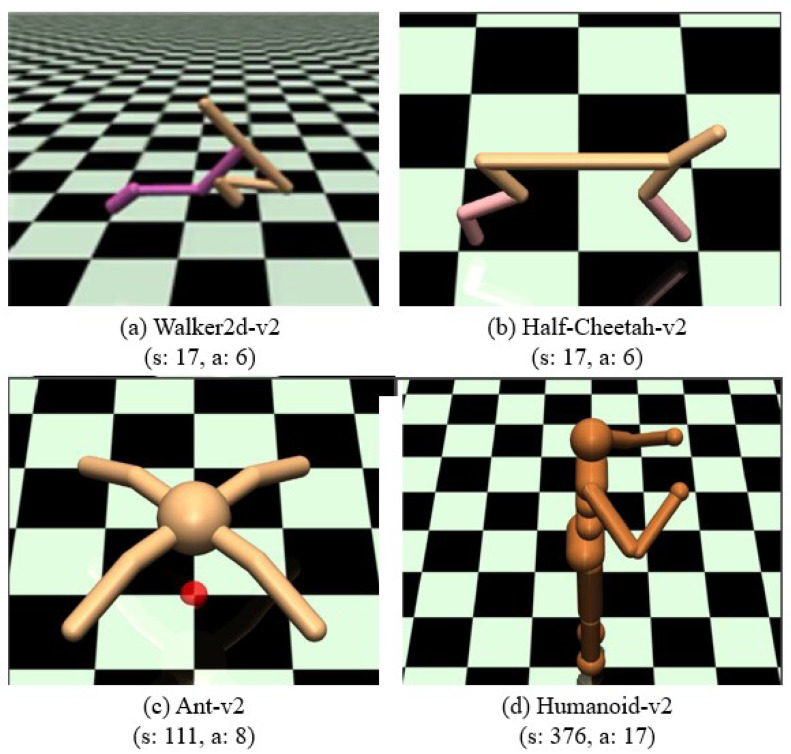
Mujoco robot learning tasks used for skill transfer learning.

To investigate the aforementioned questions, we implement the proposed algorithm on several challenging robotic locomotion tasks from the OpenAI Gym benchmark [21] and also on the rllab implementation tasks [59], which are implemented using MuJoCo [60], a 3D physics simulator with better modeling of contacts. More specifically, we build on four high-dimensional, highly nonlinear robots, including Walker2d, Half-Cheetah, Ant, and Humanoid, as shown in Figure 2. Walker2d is a planar robot with 7 links, the reward is given by $r\left(s,a\right)=v_{x}-0.005{∥a∥}_{2}^{2}$, the episode is terminated when $z_{body}<0.8$, $z_{body}>0.2$ or when |θ|>1.0, the dimension of state space and action space are 17 and 6, respectively; Half-Cheetah is a planar biped robot with 9 links, the reward is given by $r\left(s,a\right)=v_{x}-0.05{∥a∥}_{2}^{2}$, the dimension of state space and action space are 17 and 6, respectively; Ant is a quadruped robot with 13links, the reward is given by $r\left(s,a\right)=v_{x}-0.005{∥a∥}_{2}^{2}-C_{contact}+0.05$, where $C_{contact}$ penalizes contacts to the ground, and is given by $5\times 10^{-4}{∥F_{contact}∥}_{2}^{2}$, where $F_{contact}$ is the contact force, the episode is terminated when $z_{body}<0.2$ or when $z_{body}>1.0$, the dimension of state space and action space are 111 and 8, respectively; Humanoid is a human-like robot with 17 joints, including the head, body trunk, two-arms and two-legs, the reward is given as $r\left(s,a\right)=v_{x}5\times 10^{-4}{∥a∥}_{2}^{2}-C_{contact}+C_{deviation}+0.2$, where $C_{contact}=5\times 10^{-6}∥F_{contact}∥$, $C_{deviation}=5\times 10^{-3}\left(v_{y}^{2}+v_{z}^{2}\right)$, the episode is terminated when $z_{body}<0.8$ or $z_{body}>2.0$, the dimension of state space and action space are 376 and 17, respectively.

During the building of source tasks space, we consider two classes of tasks. The first class of task is the robot owns the same configuration and same physical parameters but with different tasks, which can be realized by setting different reward functions, including, Walker2d-Diff-Velocity and HalfCheetah-Diff-Velocity, the robot move forward with different velocity, where the training task set includes 100 tasks with different velocity setting, and the testing task set includes 30 tasks with different velocity setting. Ant-Random-Goal and Humanoid-Random-Dir, robot move in different directions in the 2D planar space, where the training task set includes 100 tasks with different directions, and the testing task set include 30 tasks with different directions. Another class of tasks is that the robot with different physical parameters realizes the same task. Walker2d-Diff-Params and Ant-Diff-Params, robots own different parameters, where the training task set includes 40 tasks with different physical parameters, and the testing task set includes 10 tasks with different physical parameters. To investigate the advantages of the proposed algorithm, we compare the VIB-based meta-reinforcement learning algorithm with MAML and ProMP, where the learning parameters are borrowed from the original papers.

Due to the universal approximation capability of DNNs, we parameterize the action-value function, state-value function, policy, and the latent space encoder all in feed-forward type DNNs with three hidden layers, each hidden layer owning 300 neurons. More specifically, Table 2 lists the parameters used in the algorithm for comparative evaluation.

### 4.2. Comparative Study

**Figure 3:**
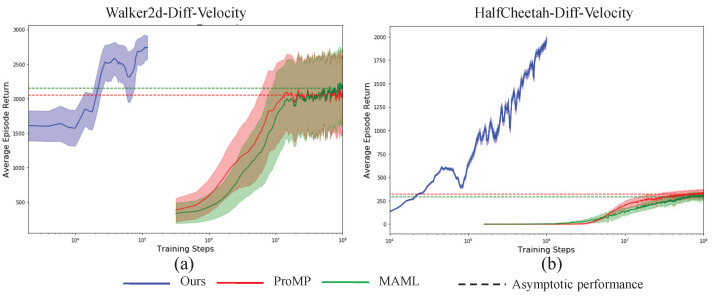
Performance comparisons for Walker2d (**a**) and Half-Cheetah (**b**) with different forward velocity transfer learning tasks.

**Figure 4:**
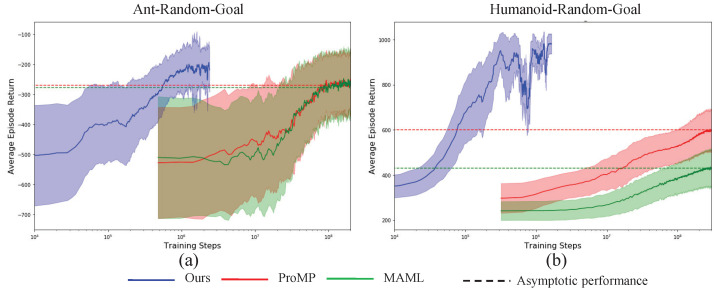
Performance comparisons for Ant (**a**) and Humanoids (**b**) with different goal location transfer learning tasks.

**Figure 5:**
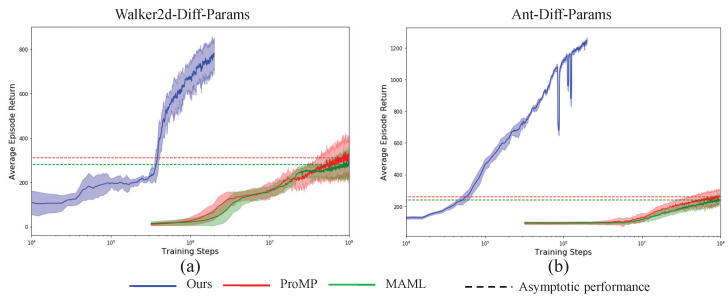
Performance comparisons for Walker2d (**a**) and Ant (**b**) with different physical parameters transfer learning tasks.

To show the efficiency and effectiveness of the presented VIB-based off-policy actor-critic algorithms, we compare our algorithm with the famous MAML and ProMP from two aspects, i.e., the sample efficiency during the training procedure, and asymptotic performance after the algorithm converges. To increase the statistical reliability of the results, all the results are obtained by taking an empirical mean and variance on 10 random seeds. The results are shown in Figure 3, Figure 4 and Figure 5. The solid lines represent the mean and the shallow part around the solid lines denotes one standard deviation, the dotted lines denote the asymptotic performance for different algorithms with a different color. From the results, the proposed algorithm achieves simultaneous big sample efficiency during training and asymptotic performance improvement. We compute the number of times for performance improvement as,

(33) $$\#\;\mathrm{Times}\;=\frac{t_{\mathrm{ReachingAsymptotic}\;}^{\mathrm{VIB}\;}}{t_{\mathrm{ReachingAsymptotic}\;}^{\mathrm{MAML}|\mathrm{ProMP}\;}},$$

where the $t_{\mathrm{ReachingAsymptotic}\;}^{\mathrm{VIB}\;}$ denotes the time moment when the proposed VIB-based meta-RL algorithm reaches the asymptotic performance of the baseline MAML or ProMP, the $t_{\mathrm{ReachingAsymptotic}\;}^{\mathrm{MAML}|\mathrm{ProMP}\;}$ denotes the time moment when the baseline MAML or ProMP algorithms reach asymptotic performance. As shown in Table 3, the sample efficiency improves by at least 200 times. In particular, for the tasks Walker2d-Diff-Velocity and Humanoid-Random-Dir, 5000 times sample efficiency improvement was achieved. We know that the Humanoid task is very challenging due to its extremely high-dimensional state space and action space, and its highly nonlinear dynamics. Therefore, the proposed VIB-based meta-reinforcement learning algorithm can realize efficient skill adaptation.

## 5. Conclusions

In this paper, we revisited the problem of efficiently adapting skills obtained with DRL methods to novel, unseen environments or tasks. By observing the facts that human beings could learn new skills very efficiently since we could draw inferences about other cases from one instance, thus the acquired skills would be adapted to new tasks and be formulated as new skills. Inspired by this idea, we argued that if the robot could extract the basic tasks and the respective basic skills from the task space, when a new task was encountered, the basic tasks and basic skills could be efficiently transferred and formulate new skills. Therefore, we took advantage of variational information bottleneck techniques and developed a latent space-based meta-reinforcement learning algorithm. The empirical results based on Mujoco benchmarking robotic locomotion tasks show that the variational information bottleneck-based meta-reinforcement learning could realize efficient skill learning and transfer. Thus, this work took a substantial step in implementing the learning-based algorithms to practical robotic skill learning. In the future, we will take a step further by applying our algorithm to much more complicated tasks, such as quadrotor aerobatic flight with quickly changing aerodynamics, and high dimensional tasks, such as tasks with image input. 

## Figures and Tables

**Table 1:** Definitions and descriptions of variables used for meta-reinforcement learning.

Symbol	Functions	Description
$p\left(T\right)$	Task distribution	Characterize a class of task
T	Task	A specific task described by MDP
S	State space	$p\left(T\right)$ shares the same state space
A	Action space	$p\left(T\right)$ share the same action space
P	Transition function space	Including varying functions,
		i.e., different robot dynamics
R	Bounded reward	Including varying reward functions,
	function space	i.e., different tasks
$\left\{T_{i}\right\}∼p\left(T\right)$	Meta-training task set	Sampling *M* tasks from source task space
$\left\{D_{T_{i}}\right\}$	Training set	The meta-training data set
$\left\{T_{j}\right\}∼p\left(T\right)$	Meta-testing task set	Sampling *N* tasks for testing
$\left\{D_{T_{j}}\right\}$	Testing set	The meta-testing data set
$e_{1:K}^{T}$	Trajectories	Trajectories for task T

**Table 2:** Hyper parameters for the proposed meta reinforcement learning algorithm.

Parameters	Symbol	Value
Optimization algorithm		Adam
Learning rate	$\eta_{\varphi },\eta_{\psi },\eta_{\theta },\eta_{\omega }$	$3\times 10^{-4}$
Discounting factor	γ	0.99
Entropy weighting	1α	5
Lagrange multiplier	*β*	0.1
Information constraint	*I_c_*	1.0
Number of the hidden layers	*Q*, *V*, *π*	3
	*E*	3
Number of neurons in each layer	*Q*, *V*, *π*	300
	*E*	200
Nonlinear activator		ReLU
The maximum path length		200
Samples for each mini-batch	*M*	256
The frequency for target network updating	*τ*	1000

**Table 3:** Quantitative studies the proposed meta reinforcement learning algorithm.

Tasks	Performance Improvement (# Times )
Walker2d-Diff-Velocity	≈5000
HalfCheetah-Diff-Velocity	≈4000
Ant-Forward-Back	≈2500
Humanoid-Random-Dir	≈5000
Walker2d-Diff-Params	≈200
Ant-Diff-Params	≈200

## Data Availability

Not applicable.
